# Development of an aerogenous *Escherichia coli* infection model in adult broiler breeders

**DOI:** 10.1038/s41598-021-98270-8

**Published:** 2021-10-01

**Authors:** Sofie Kromann, Rikke Heidemann Olsen, Anders Miki Bojesen, Henrik Elvang Jensen, Ida Thøfner

**Affiliations:** 1 Department of Veterinary and Animal Sciences, Faculty of Health and Medical Sciences, University of Copenhagen, Ridebanevej 3, 1870 Frederiksberg C, Denmark; 2 DanHatch Denmark A/S, Rugerivej 26, 9760 Vrå, Denmark

**Keywords:** Bacterial infection, Infection, Experimental models of disease

## Abstract

*Escherichia coli* constitutes an immense challenge to the poultry industry due to its devastating effect on productivity, mortality, and carcass condemnations. To aid future studies on disease mechanisms and interventions, an aerogenous infection model was established in adult broiler breeders. Hens (n = 120) were randomly allocated into six groups receiving either aerosolised *E. coli* or vehicle, or intratracheal *E. coli* or vehicle. Replication of aerosol inoculation was performed on distinct days. Alternating euthanasia time points were predetermined in order to evaluate the progression of the disease. All animals were thoroughly necropsied, and bacteriological samples were collected as well as tissues for histopathology. Birds inoculated with *E. coli* exhibited clinical signs and developed characteristic gross and histopathological lesions of colibacillosis, including splenic fibrinoid necrosis, folliculitis, polyserositis and impaction of parabronchi with fibrinoheterophilic exudate and necrotic debris, as well as positive in situ localisation of intralesional *E. coli* by immunohistochemistry. This study presents a successful development of a discriminative colibacillosis model through aerosol inoculation of adult broiler breeders. Gross and histopathological lesions characteristic of colibacillosis were established in two independent experiments.

## Introduction

Avian colibacillosis represents an ongoing and major challenge to the poultry industry due to the role of *Escherichia coli* as the commonest bacteriological pathogen with substantial economic consequences^1,2^. Decades of studies have documented *E. coli*’s devastating contributions to impaired animal welfare, carcass condemnation, antibiotic use and mortality^3–9^.

The lesions inflicted by *E. coli* in poultry are typically extraintestinal, often collectively referred to as colibacillosis and manifest in a number of ways including cellulitis, omphalitis and yolksacculitis, peritonitis, perihepatitis, pericarditis, airsacculitis, oophoritis, salpingitis, arthritis and osteomyelitis^1,10^.

As a natural inhabitant of the intestinal tract, *E. coli* is continuously passed to the environment through droppings^10,11^ providing a potential source for dissemination through dust within housing facilities with numbers as high as 10^6^ colony forming units (CFU) per gram being reported^12^. Transmission occurs horizontally, either directly or indirectly, as well as vertically from breeders to progeny^1,13^. The pathogenesis of avian colibacillosis is often attributed to the respiratory route^10,14^, with ascending infections via the oviduct, entrance through a compromised skin barrier and translocation of bacteria from the intestinal tract as additional important routes of infection^15–17^.

Experimental in vivo models provide an essential tool for understanding disease processes and mechanisms and are indispensable in the development of effective preventive strategies and therapeutic agents. A wide range of models attempting to mimic colibacillosis in controlled settings has been developed in domestic poultry with varying degrees of success and invasiveness, e.g*.*, inoculation intratracheally, directly into air sacs, intra-uterine, subcutaneously, intravenously, as well as through aerosols in layers and small chickens, and intraperitoneally^14–16,18–21^.

When critically evaluating an experimental animal model, concepts of validity become essential, i.e*.*, especially the predictive validity of the model, addressing how well the results obtained predicts the outcome in the situation of interest, e.g*.*, if the efficacy of a treatment in the model correlates with the outcome of treatment in the field. Other important validity concepts are face validity and construct validity, with the former reflecting how well the model mirrors the actual condition, e.g*.*, if lesions appear pathomorphologically similar to that of field cases and if clinical signs are similar to those of spontaneous disease. Construct validity focuses on the underlying mechanisms behind the disease—how well do the pathogenesis and disease mechanisms in the model adhere to that of spontaneous disease^22^. Collectively, models with high validity on these crucial concepts represent a discriminative model, i.e., in the case of an infection model, the route of infection, agent, disease progression, symptoms, pathology and immunocompetence are highly alike those of the true disease^23^.

Despite a broad range of colibacillosis models being available, their value is often somewhat debatable due to the failure of proper reporting of basic concepts associated with the study design—e.g*.*, the proper use of randomisation, blinding and even basic details about the animals, inoculum and interventions. Consequently, this impairs the chance of reproducing the study as well as evaluating the quality and validity of the model—a problem well known in animal experiments^24,25^.

The aim of the present study was to develop and adequately describe a discriminative aerogenous model of avian colibacillosis in adult broiler breeders.

## Materials and methods

### Animals and housing

Ross 308 hens (n = 120) at 29 weeks of age were obtained from SweHatch, Sweden, distributed into six pens utilising randomisation and acclimatised for one week. The animals were housed in groups of 20 in 8.64 square meter coops with wood shavings as bedding and turf dust-baths, straw, hay, shelves, and perches as enrichment. Feed was provided once a day (155/g/hen) and consisted of commercial wholefood for egg-laying hens supplemented with sunflower seeds, wheat- and barley kernels, and fish meal. Water was available ad libitum, and three hooded cat litter boxes were offered as nests in each coop. A light–dark cycle of 12 h light, including 30-min dim-phases, and 12 h of darkness was maintained. The study was conducted during the summer with morning temperatures ranging between 19.4 and 26.6 °C within the facilities. Bodyweight (BW) was registered prior to inoculation and euthanasia, whereas egg-count was obtained daily on pen-level following a standardised time schedule. The animals had been vaccinated according to the programme presented in Table 1, lacking prophylactic interventions targeting *E. coli*.

**Table 1:** Vaccination programme.

W	Vaccination (target)
5–9 d	Paracox-8 Vet. (Coccidia)
4	Nobilis IB Ma5 Vet. (IB)
6	Nobilis Gumboro D78 Vet. (IBD)
8	Nobilis 4/91 Vet. (IB)
10	AviPro Thymovac (CAV)
12	AviPro AE (AE)
14	Nobilis Gumboro D78 Vet. (IBD)
16	Nobilis IB Ma5 Vet. (IB)

Note the absence of *E. coli* prophylaxis.

*AE* avian encephalomyelitis, *CAV* chicken anaemia virus, *d* day, *IB* infectious bronchitis virus, *IBD* infectious bursal disease, *W* week of life.

### Experimental design

On the day of arrival, each coop containing randomly allocated animals (n = 20/pen) was assigned to one of six study groups by randomisation. The study groups were as follows: Aerosol_1 and Aerosol_2 inoculated with aerosolised *E. coli* at alternate time points, utilising a specialised aerosol-chamber described below, concomitant with appropriate controls (Control_1 and Control_2, respectively) receiving aerosolised vehicle, as well as a group inoculated intratracheally with either *E. coli* (IT) or vehicle (IT_control). The IT group was included as a positive control and was inoculated simultaneously with Aerosol_1 with a CFU dose-matched to the estimated CFU inhaled by the aerosol groups.

**Figure 1:**
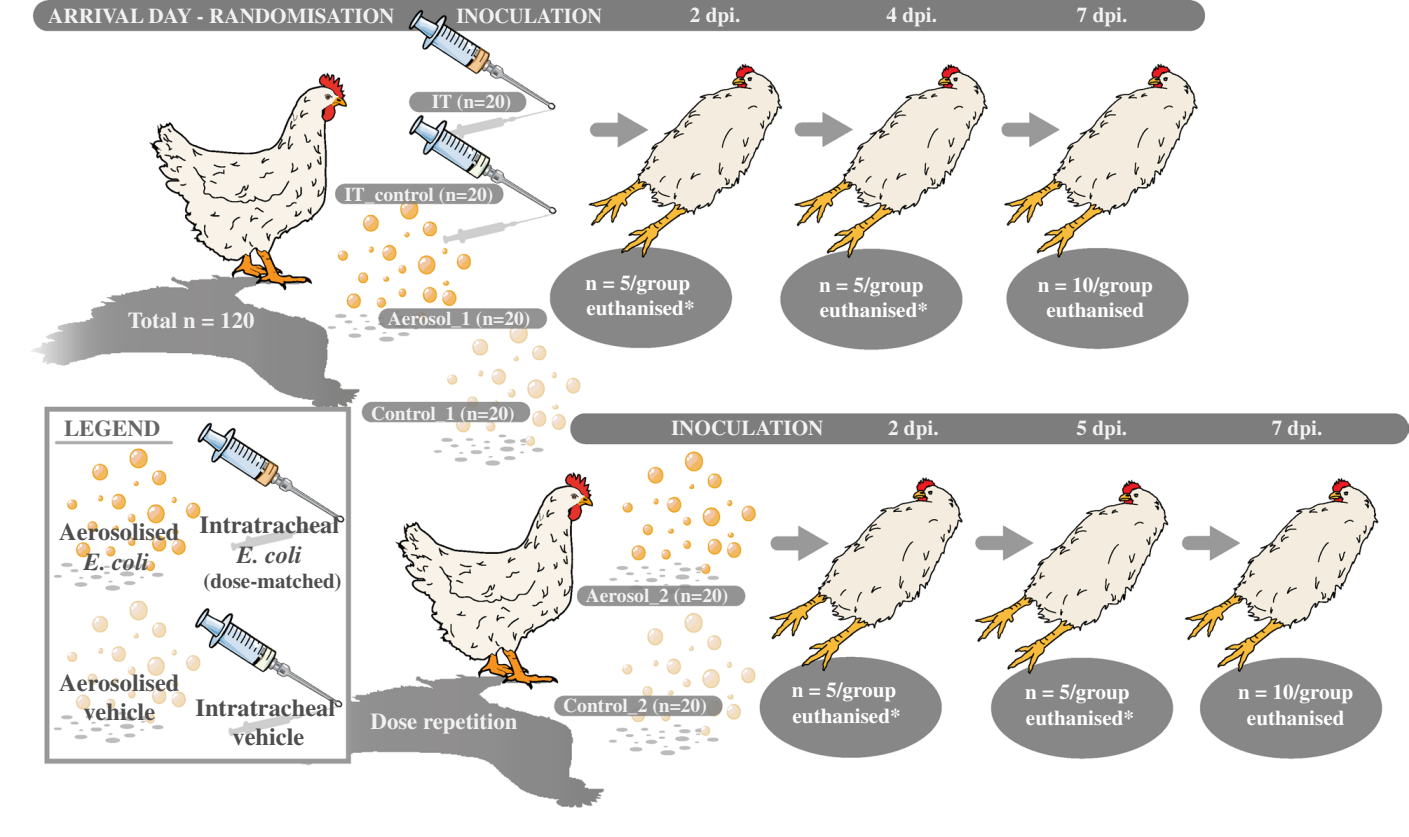
Study outline. Broiler breeder hens (n = 120) were allocated by randomisation to one of six groups and acclimatised for one week, and subsequently inoculated and euthanised according to the displayed timeline. *Animals were designated for euthanasia by randomisation, and deviations from the schedule only occurred if animal welfare considerations made premature euthanasia necessary. Aerosol inoculation was replicated at alternating days to evaluate the reproducibility of the results obtained through this route of infection. An intratracheally inoculated group was initially included as a positive infection control. *dpi* days post-infection; *n* number; *IT* intratracheal.

Two days following inoculation of Aerosol_1 and the IT group, Aerosol_2 was exposed to an identical challenge in an attempt to replicate the initial outcome. In order to evaluate the progression of the disease, the animals were designated for euthanasia according to the schedule presented in Fig. 1. Appointment to the different days of euthanasia was done by randomisation. Divergence from the outlined schedule only took place if animal welfare considerations required prescheduled euthanasia.

### Aerosol chamber

**Figure 2:**
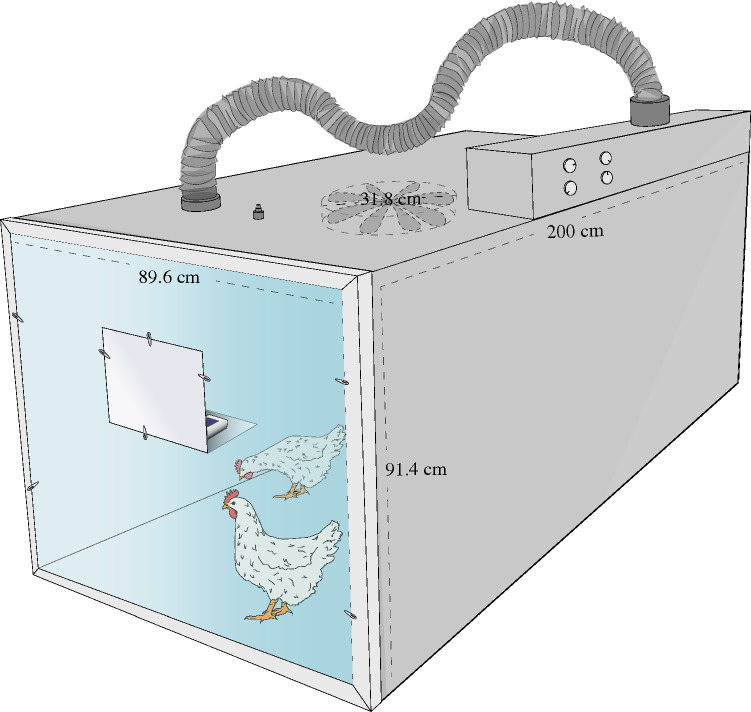
Aerosol chamber. Illustration of the aerosol-chamber utilised for inoculation. Internal measurements were 91.4 × 89.6 × 200 cm, a fan with a diameter of 31.8 cm was located in close proximity to the centre of the chamber, and an inlet-system for aerosols and an outlet system with an inbuilt HEPA-filter were part of the construction. The chamber was constructed in hard plastic (14 mm) except from one end consisting of a plexiglass-gate, hereby allowing continuous monitoring of animals within the chamber. A hatch was created in the plexiglass gate, in close proximity to an internal shelf used for the AeroCollect air-samplers and, when animals were kept within the chamber, a datalogger for temperature and air humidity.

The aerosol chamber consisted of a 91.4 × 89.6 × 200 cm (internal measurements) box made of 14 mm hard plastic, except at one end consisting of a gate made of plexiglass allowing continuous animal monitoring (Fig. 2). All assemblies, as well as hinges and internal screws, were enclosed with grout, and the gate was made airtight using a rubber gasket to ensure an adequate seal. A metal fan measuring 31.8 cm in diameter and running with a constant speed of approximately 2700 rpm was attached to the chamber ceiling in close proximity to its centre. The chamber was constructed with an inlet system for aerosols and an outlet system equipped with a HEPA-filter. Prior to animal inoculation, the obtainable CFU/L air within the chamber, and reproducibility thereof, was examined along with the bacterial intra-chamber distribution through a series of test runs. For this purpose, the AeroCollect (Force Technology, Denmark) air-samplers was secured on a metal shelf, attached to the inside of the plexiglass gate, and utilised for approximate CFU measurements by quantitative PCR (qPCR). The measurements were conducted in duplicates, besides from one time point where 17 air-samplers were distributed throughout the chamber in order to assess the possible intra-chamber variation. Analyses of all obtained air samples were executed by qPCR at the AeroCollect by FORCE Technology’s laboratory, Denmark, with a ct-value set at 26. In addition to the AeroCollect air-samplers, MacConkey agar plates were distributed in the chamber to further analyse the bacterial distribution. The plates were positioned as follows: (1) each side of the front part of the chamber, each side of the middle part and each side of the back part, and (2) similar position to (1) but positioned at a height of approximately 35 cm. Repetition of (1) was done three times, whereas (2) was performed twice. For all the runs containing bacteria, the inoculum was prepared according to the procedure described below, examined by optical density (OD) and serially diluted to determine the CFU/mL. To aerosolise the inoculum, an Omron Ultrasonic Nebulizer NE-U17 nebuliser was used with all the following parameters set to 10: nebulisation time (minutes), airflow, and nebulisation volume. Throughout the study, the nebuliser unit was prepared according to the manufacture’s description. Following nebulisation, five minutes of ventilation was upheld prior to opening the aerosol-chamber as a safety measurement. Between test-runs, the chamber and aerosolisation equipment were cleaned with either Virkon S, prepared according to the manufacturer’s instruction, or 70% ethanol. Each day of sample collection, the test-runs were initiated with sterile H_2_O whilst gathering air samples. This was done to evaluate the cleaning procedure to exclude bacterial carryover between groups. Following the initial run, the cleaning procedure of the internal surfaces was further evaluated by collection of bacterial samples with cotton swabs streaked onto MacConkey agar.

### Strain and inoculum preparation

The *E. coli* strain, ST117 E44 (accession number LXWV00000000.1), utilised for inoculation was originally isolated from a clinical case of colibacillosis and was provided by the Section for Veterinary Clinical Microbiology at the University of Copenhagen. The inoculum was prepared as follows: *E. coli* ST117 E44, stored at -80 °C, was streaked onto blood agar base supplemented with 5% bovine blood (BA) and incubated at 37 °C overnight. Fifty mL sterile centrifuge tubes containing 10 mL of Lysogeny broth (LB) each were then prepared by adding a single colony to each tube, followed by vortex-mixing, and incubation on shake (125 rpm) at 37 °C for 19 h, thereby providing an overnight culture. Subsequently, 250 mL glass Erlenmeyer flasks containing 50 mL of LB each were prepared by adding 500 µL of overnight culture to each of the flasks, which were incubated at 37 °C and shake (125 rpm) for four hours providing an exponential phase inoculum. Immediately following four hours, the inoculum was placed on ice, OD was measured, and ten-fold dilutions were made in duplicates and plated onto Lysogeny agar, using glass-beads, for CFU confirmation. Sterile vehicle (LB) for sham-inoculation of the control groups was kept under conditions similar to the inoculum.

### Aerosol challenge

Aerosol inoculation was conducted as follows: 10 birds from each group were placed in the aerosol-chamber concurrently for a total of 15 min, subdivided into 10 min of aerosolisation and 5 min of ventilation to clear the chamber prior to evacuation of birds. The Omron Ultrasonic Nebulizer NE-U17 settings were as described above. Aerosol controls were inoculated firstly to avoid carryover of *E. coli* to the control groups, and, additionally, a “pre-run” with sterile saline was conducted prior to vehicle exposure to confirm the absence of *E. coli* within the setup. During all series of inoculation, as well as the pre-runs, air samples were collected in duplicate to monitor the *E. coli* levels, and a datalogger was utilised for continuous monitoring of temperature and humidity inside the chamber. Air samples were analysed according to the above description, and animals were returned to their coops immediately following aerosol exposure.

### Intra-tracheal challenge

Air samples collected during the aerosol-chamber preparations, along with a previously reported minute ventilation rate of 261 mL/min/kg White Leghorn hen^26^ and the mean BW of the animals, were used to calculate the dose assumed to be inhaled by the aerosol inoculated animals in an attempt to dose-match the groups. The IT inoculum was prepared by diluting the inoculum, prepared for aerosolisation, to a CFU/mL equal to the estimated inhaled CFU, whereafter, syringes were prepared containing 1 mL each. Syringes containing sterile vehicle (LB) were prepared for the IT controls, and all syringes were placed on ice immediately after preparation. Ten-fold serial dilutions were plated as described above for CFU confirmation. Inoculation of the animals was carried out as follows: an assistant briefly fixated each hen whilst gently forcing the beak to open, allowing a veterinarian to introduce the syringe, prepared with a buttoned steel cannula, carefully into the trachea for deposition of the inoculum directly into the airways.

### Euthanasia, post-mortem examination and sampling

The animals were euthanised as illustrated in Fig. 1 by cervical dislocation directly following the induction of unconsciousness by blunt head trauma. Gross evaluation of the pathology was performed in a randomised and blinded manner following a systematic evaluation scheme ensuring a full necropsy and preventing a solely problem-oriented approach. Prior to necropsy, post-mortem BW was registered on a balance with an uncertainty of ± 0.5 g. During necropsy, the liver, spleen, and right lung were systematically weighed on an analytical balance (readability: 0.01 g, uncertainty: ± 0.02 g), thereby allowing the comparison of the organ to BW-ratios between groups. Overall lesion scores were calculated by dividing the sum of lesions in each group with the number of birds within the respective group. The following bacteriological samples were obtained systematically from all animals: right thoracic air sac, liver (through the visceral surface), peritoneum, spleen, lumen of salpinx (infundibulum and middle magnum), femoral bone marrow, the left lung, and the middle part of trachea. Sampling of the left lung was performed by removal with sterilised instruments, followed by stomaching in one mL 0.9% sterile saline for approximately one minute, and subsequent plating from the solution with a cotton swab onto BA. The bone marrow was accessed by breakage of the femoral head at the level of the diaphysis. Deviation from the weight registration of the right lung was only made in case of accidental contamination of the left lung rendering this unsuitable for bacteriological sampling. Tissue samples for histopathology were collected from the right lung, middle of trachea, spleen, liver, infundibulum, magnum, and ovary were collected and immediately suspended in 10% formalin. A semi-quantitative scoring system of crop-content was applied as follows: no palpable content was graded as 0, presence of noticeable content as 1, and filled as 2.

### Microbiology

Bacteriological sampling was performed using either sterile standard wooden cotton-swabs or steel cotton-swabs, depending on the sampling site. All swabs were subsequently plated on BA plates. Thereafter, the plates were incubated for approximately 24 h at 37 °C, after which they were examined visually for *E. coli* growth by skilled microbiologists. Plates were considered positive if exhibiting pure growth of *E. coli* or being clearly dominated by *E. coli*. Tentative *E. coli* isolates were sub-cultured onto BA and incubated for 24 h at 37 °C. Species identification of *E. coli* was confirmed by PCR according to Chen and Griffith^27^, using boiling lysates as DNA templates. All confirmed *E. coli* isolates were subsequently characterised by Multiple-Locus Variable-Number Tandem-Repeat Analysis (MLVA) according to Camelena et al.^28^, except DreamTaq (Fermentas, Thermo Scientific, Roskilde, Denmark) replaced Qiagen multiplex kit. An isolate of *E. coli* ST117 used in the infection trials was included in the MLVA typing assay, and all MLVA-typed strains were visually determined to have band patterns identical to or different from this strain, respectively.

### Histopathological evaluation

Following fixation in 10% formalin, hepatic, splenic and pulmonary tissue from animals euthanised at 2 dpi and 7 dpi was embedded in paraffin and cut into 2–4 μm sections, which were stained with haematoxylin and eosin (H&E) for morphological evaluation^29^. The tissues were evaluated systematically at 200× magnification or higher, and lesions were recorded. In a subset of sections with lesions, the presence of *E. coli*-bacteria was confirmed by immunohistochemistry as previously described^30^.

### Ethics

The study was approved by the Danish Animal Experiments Inspectorate under the Danish Ministry of Environment and Food, and all animal procedures were performed in accordance with this approval (license no. 2019-15-0201-01611) and with ARRIVE guidelines. The licence granted, and guidelines hereof, is in agreement with the Danish law on animal experiments and the EU directive 2010/63. Predefined humane endpoints were determined, and animals were observed every 30 min for the initial six hours following inoculation, subsequently at maximally eight-hour intervals for three days following inoculation with an increased frequency in case of clinical signs in any of the groups. If clinical signs were present, e.g., ruffled feathers, depression, anorexia, lethargy or dyspnoea, the bird was either treated with 0.1 mg/kg buprenorphine and observed with increased frequency or euthanised.

### Statistics

All statistical analyses were performed utilising GraphPad Prism version 9.0.1 for Mac OS X (GraphPad Software, Inc., La Jolla, USA) (www.graphpad.com). The normality of continuous data was assessed by the Kolmogorov–Smirnov normality test. If the data did not follow a normal distribution, a logarithmic transformation was performed to achieve normality if possible. In cases of parametric data, comparison was made using either a T-test or one-way analysis of variance (ANOVA), as appropriate, using Tukey's multiple comparisons test for post hoc analyses. Non-parametric data and ordinal data were analysed utilising Mann–Whitney U-test or Kruskal–Wallis according to the number of groups compared. Dunn’s multiple comparisons test was used as post hoc test in case of the latter. All parametric data is presented with mean ± SD, whilst the median is available for non-parametric and ordinal data. Logarithmically transformed data is presented as original data. The significance level was set at *p* < 0.05. Each randomisation-step throughout the study was performed using the rand() function Microsoft Excel for Mac version 16.52 (www.microsoft.com).

### Ethical approval

The Danish Animal Experiments Inspectorate under the Danish Ministry of Environment and Food approved the study, and all animal procedures were performed in accordance with the approval (License No. 2019-15-0201-01611).

## Results

### Aerosol chamber evaluation

An inoculum concentration of more than 1 × 10^5^ CFU/L air (average 6 × 10^5^ CFU/L) was confirmed as reproducible at four different time points on three separate days (Table 2). No apparent intra-chamber variation was detected, and swabs collected following the cleaning procedure were sterile, and air samples collected during H_2_O-runs, following the cleaning procedure, did not contain *E. coli*.

**Table 2:** Chamber evaluation.

Test run day	Nebulised substance	CFU/mL	CFU/L air
1	Sterile H_2_O	0	0
1	*E. coli* inoculum	2.47 × 10^9^	3.04 × 10^5^
1	Sterile H_2_O	0	0
1	*E. coli* inoculum	2.47 × 10^9^	6.72 × 10^5^
2	Sterile H_2_O	0	0
2	*E. coli* inoculum	1.4 × 10^9^	9.68 × 10^5^
3	Sterile H_2_O	0	0
3	*E. coli* inoculum	1.94 × 10^9^	4.5 × 10^5^

The objective was to determine the obtainable CFU/L air within the chamber and, additionally, the distribution and reproducibility hereof. Throughout all the test-runs, the Omron Ultrasonic Nebulizer NE-U17, used for aerosolisation, had the following set to 10: airflow, nebulisation volume and nebulisation time. All runs were succeeded by five minutes of ventilation of the chamber for safety reasons. AeroCollect air-samplers were utilised to determine the CFU/L air in duplicate. Quantitative PCR, performed in duplicates, was used to determine the *E. coli* CFU/L air. The mean of the measurements is presented.

*CFU* colony forming unit, *E. coli* *Escherichia coli.*

### *E. coli* exposure

Air samples obtained during inoculation of Aerosol_1 and Aerosol_2 revealed an exposure of 1.45 × 10^5^ CFU/L corresponding to an estimated inhaled dose of 1.9 × 10^7^ CFU and 1.89 × 10^7^ CFU, respectively, whilst the IT group received 7.42 × 10^6^ CFU (Table 3). During sham-inoculation of aerosol controls, i.e., Control_1 and Control_2, *E. coli* was not detected in the air samples. All animals were clinically unaffected immediately following inoculation. Temperature levels in the aerosol-chamber during exposure of the Aerosol_1 and Control 1 group ranged from approximately 24.3 °C to 27.8 °C, whilst these numbers were 30.8 °C to 32.1 °C during exposure of Aerosol_2 and Control_2, respectively. During peak exposure times in all runs, the relative humidity within the chamber reached levels above 80%.

**Table 3:** Exposure.

Group	Inoculum	Route of inoculation	Volume or exposure time	CFU/mL	CFU/L air*	Dose (CFU)
IT	*E. coli*	Intra-tracheal	1 mL	7.42 × 10^6^	**–**	7.42 × 10^6^
IT_control	LB	Intra-tracheal	1 mL	0	–	0
Aerosol_1	*E. coli*	Aerosols	10 (15)^a^ min	2.77 × 10^9^	1.45 × 10^6^	1.9 × 10^7b^
Control_1	LB	Aerosols	10 (15)^a^ min	0	0	0
Aerosol_2	*E. coli*	Aerosols	10 (15)^a^ min	2.33 × 10^9^	1.38 × 10^6^	1.89 × 10^**7**b^
Control_2	LB	Aerosols	10 (15)^a^ min	0	0	0

*Mean of the obtained air samples collected in duplicate at two rounds of exposure of n = 10 birds.

^a^The time period provided in brackets encompasses the total time-length the animals spend in the aerosol chamber, i.e., exposure and ventilation time.

^b^Anticipated inhaled dose.

*CFU* colony forming unit, *E. coli Escherichia* coli, *IT* intratracheal, *LB* Lysogeny broth, *min.* minutes.

### Clinical observations and preterm mortality

One animal, belonging to the IT_control group, was euthanised prior to the study due to severe chronic pododermatitis unrelated to this experiment. In all groups exposed to *E. coli*, preterm euthanasia was necessary (Table 4) due to severe clinical signs characterised by, e.g*.*, severe depression, dyspnoea, lethargy and/or anorexia refractory to pain relief. A single animal, belonging to the Aerosol_2 group, died spontaneously, whereas the remaining animals were euthanised. None of the IT inoculated animals receiving *E. coli* survived for the entire predetermined period as all had to be euthanised preterm, primarily due to dyspnoea with severely compromised breathing and abnormal respiratory sounds. Regardless of the exposure route, all control animals receiving sterile vehicle were euthanised according to the predetermined schedule, and none of the animals in the control groups developed any clinical signs. No differences in BW (Supplementary Table S1) or egg count occurred between groups during the study.

**Table 4:** Time of euthanasia following inoculation.

Dpi	IT	IT_control	Aerosol_1	Control_1	Dpi	Aerosol_2	Control_2
2	8	5	8	5	2	7^a^	5
(3)	2	0	0	0	5	5	5
4	6	5	5	5	7	8	10
(6)	4	0	0	0			
7	–	9*	7	10			

Number of birds in each group euthanised at the given dpi. Days presented in brackets were not prescheduled days for euthanasia, however, birds were culled due to severe clinical signs.

^a^From the Aerosol_2 group, one animal died spontaneously from severe, acute septicaemia confirmed by florid growth of *E. coli* from the bone marrow.

*The intratracheal control group contained a lower overall number of birds, as one had to be euthanised shortly following arrival due to a severe case of chronic pododermatitis.

*dpi.* days post-infection, *IT* intra-tracheal.

### Gross pathology

**Figure 3:**
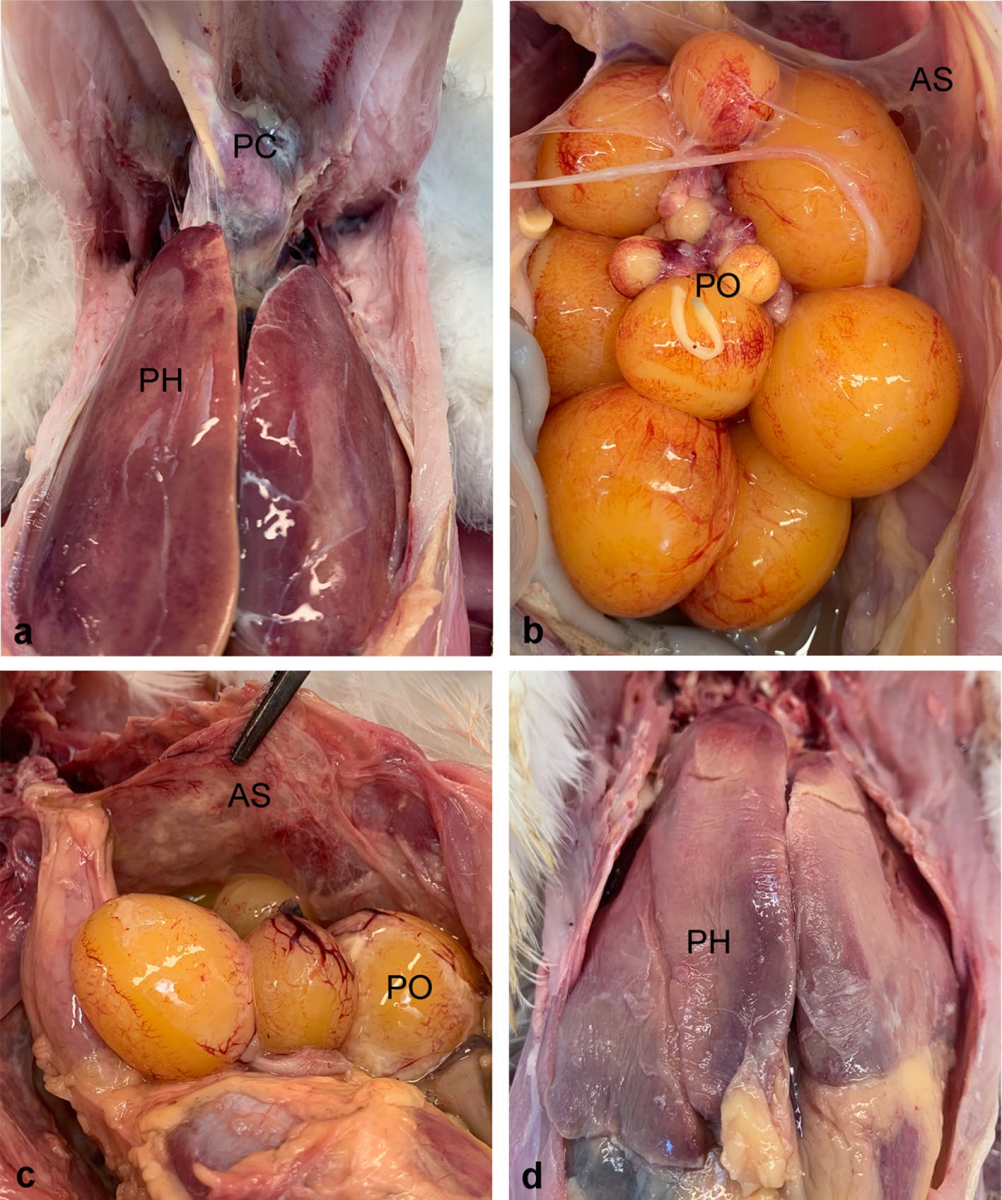
Gross lesions. (**a**) Acute fibrinous perihepatitis (PH) and fibrinopurulent pericarditis (PC) in an Aerosol_1 bird euthanised 2 dpi (**b**) Another Aerosol_1 animal euthanised 2 dpi with multifocal fibrinopurulent perioophoritis (PO). Note the opaque and milky appearance of the remnants of the thoracic air sac (AS). (**c**) A case of severe fibrinopurulent airsacculitis (AS) with prominent hyperaemia and congestion. Note the fibrinopurulent perioophoritis (PO). The bird belonged to the Aerosol_2 group and was euthanised preterm (2 dpi) in accordance with the predefined humane endpoint. (**d**) Liver from the same bird as (**c**) with a fibrinous perihepatitis (PH) and the underlying hepatic parenchyma appearing dry indicating necrosis. *dpi* days post-infection.

At necropsy, infected birds had developed lesions (Table 5) such as tracheal inflammation, with or without discharge, consolidated lungs, some with overlaying fibrinopurulent material, changes to the air sacs, fibrinous or fibrinopurulent perihepatitis (Fig. 3A and D) and/or pericarditis as well as exudative peritonitis of varying degree and exudate type. Also, the reproductive organs exhibited gross changes, i.e., purulent and/or fibrinous oophoritis (Fig. 3B and C) and alterations to salpinx characterised by hyperaemia, oedema and, in some cases, purulent salpingitis. Vascular changes on the ovarian follicles were recorded amongst both the infected and the control birds.

**Table 5:** Necropsy overview.

	IT (n = 20)	IT_control (n = 19)^a^	Aerosol_1 (n = 20)	Control_1 (n = 20)	Aerosol_2 (n = 20)	Control_2 (n = 20)
**Tracheal changes**						
2 dpi	7/8	1/5	6/8	0/5	2/7	0/5
3, 4 and 5 dpi	4/7	2/5	3/5	1/5	2/5	0/5
6 and 7 dpi	4/4	0/9	3/7	0/10	0/8	0/10
**Pulmonary changes**						
2 dpi	8/8	0/5	6/8	0/5	3/7	0/5
3, 4 and 5 dpi	7/7	0/5	3/5	0/5	3/5	0/5
6 and 7 dpi	3/4	1/9	4/7	2/10	2/8	1/10
**Airsacculitis**						
2 dpi	6/8	0/5	4/8	0/5	3/7	0/5
3, 4 and 5 dpi	7/8	0/5	0/5	0/5	0/5	0/5
6 and 7 dpi	3/4	0/9	2/7	0/10	1/8	0/10
**Pericarditis**						
2 dpi	2/8	0/5	3/8	0/5	2/7	0/5
3, 4 and 5 dpi	2/8	0/5	0/5	0/5	1/5^b^	0/5
6 and 7 dpi	1/4^b^	0/9	0/7	0/10	0/8	0/10
**Perihepatitis**						
2 dpi	2/8	0/5	4/8	0/5	2/7	0/5
3, 4 and 5 dpi	3/8	0/5	0/5	0/5	0/5	0/5
6 and 7 dpi	0/4	0/9	0/7	0/10	0/8	0/10
**Peritonitis**						
2 dpi	3/8	1/5	5/8	1/5	1/7	0/5
3, 4 and 5 dpi	3/8	0/5	0/5	0/5	1/5	1/5
6 and 7 dpi	0/4	0/9	0/7	0/10	0/8	0/10
**Perioophoritis**						
2 dpi	4/8	0/5	4/8	0/5	1/7	0/5
3, 4 and 5 dpi	2/8	0/5	0/5	0/5	2/5	1/5
6 and 7 dpi	0/4	0/9	0/7	1/10	0/8	0/10
**Ovarian follicles**						
2 dpi	5/8	1/5	4/8	0/5	4/7	0/5
3, 4 and 5 dpi	2/8	1/5	2/5	0/5	3/5	2/5
6 and 7 dpi	2/4	3/9	6/7	6/10	3/8	5/10
**Salpinx alterations**						
2 dpi	6/8	0/5	5/8	0/5	4/7	0/5
3, 4 and 5 dpi	3/7	1/5	0/5	0/5	3/5	2/5
6 and 7 dpi	2/4	1/9	2/7	1/10	0/8	0/10
Overall lesion score	4.55	0.63	3.3	0.6	2.15	0.6

^a^One animal in the IT_control group was euthanised due to a chronic pododermatitis unrelated to the study.

^b^Focal pericarditis. All presented pathological parameters were evaluated grossly. Tracheal changes were registered in case of hyperaemia and/or presence of exudate (mucoid, purulent and/or fibrinous), pulmonary changes if consolidation, oedema and/or exudate occurred, and airsacculitis if hyperaemia, opaqueness and/or exudate were present. Pericarditis, perihepatitis, peritonitis and perioophoritis were registered if purulent and/or fibrinous exudate occurred, whereas alterations to salpinx were noted in case of hyperaemia, oedema and/or exudate. Furthermore, vascular changes to the ovarian follicles were registered if hyperaemia or congestion appeared.

*dpi* days post-infection, *IT* intratracheal, *n* number.

**Figure 4:**
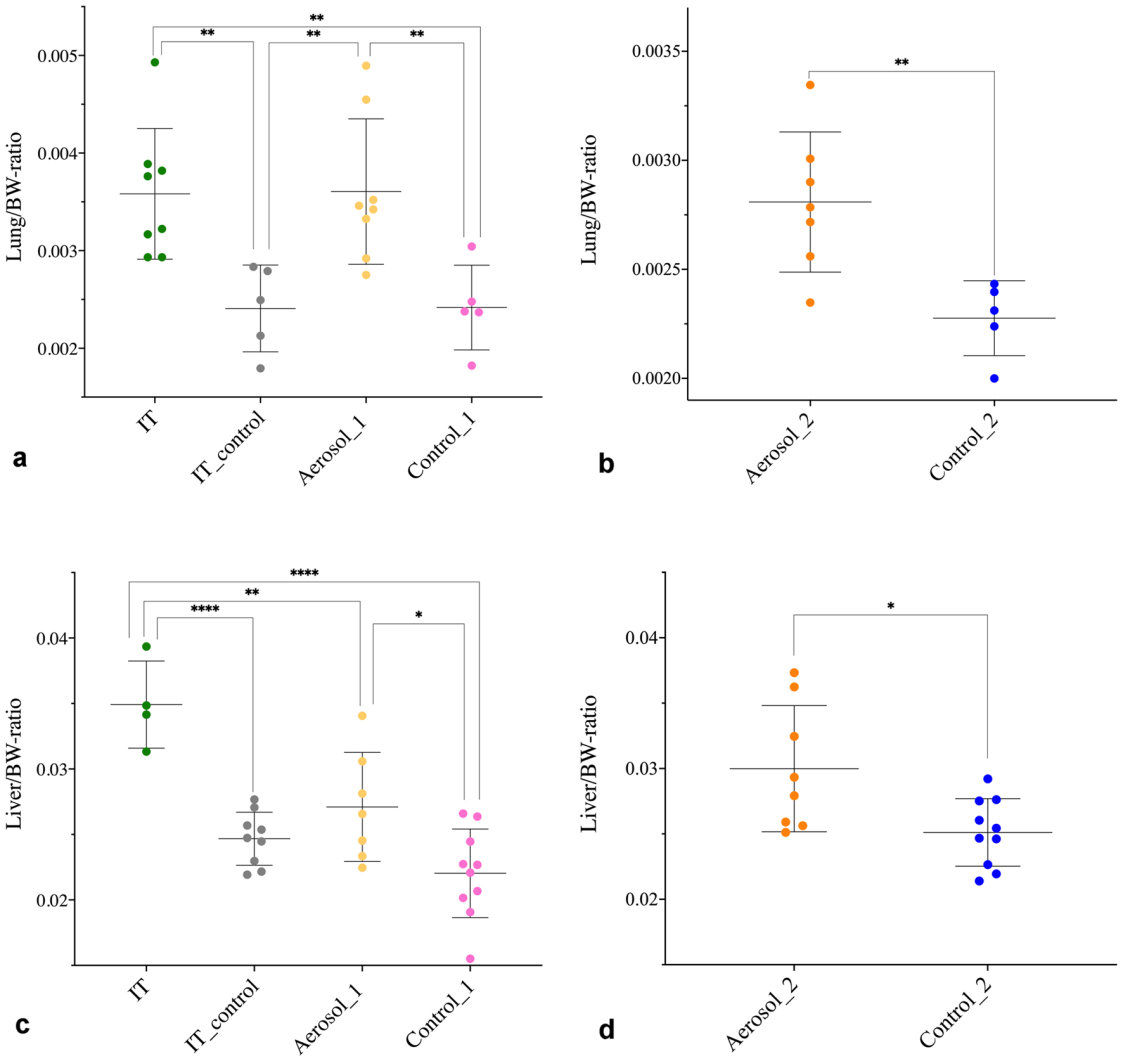
Organ/BW-ratio. (**a** and **b**) presents the lung/BW-ratio at euthanasia 2 dpi. In (**c** and **d**), the liver/BW-ratios at 6 and 7 dpi are displayed. Data are presented with mean ± standard deviation. Data presented in (**a** and **c**) were analysed using ANOVA and Tukey’s multiple comparison test. In a) the data were log-transformed to obtain normality prior to analyses. (**a**) Aerosol_1 vs Control_1, **p = 0.0065; Aerosol_1 vs IT_control, **p = 0.0058; IT vs Control_1, **p = 0.007; IT vs IT_control, **p = 0.0062. In c) Aerosol_1 vs Control_1, *p = 0.019; IT vs Control_1, ****p < 0.0001; IT vs IT_control, ****p < 0.0001; IT vs Aerosol_1, **p = 0.0036. Data displayed in (**b** and **d**) were analysed utilising a T-test. In (**b**) **p = 0.0074 and (**d**) *p = 0.0142. *BW* bodyweight; *dpi* days post-infection; *IT* intra-tracheal.

A difference in the lung/BW-ratio was evident between *E. coli* inoculated groups and the control groups at 2 dpi, with the former having a significantly higher lung/BW-ratio compared to the latter groups (Fig. 4A and B). A difference was not detectable at other euthanasia points except between the IT group and the Control_1 at 4 dpi. Liver/BW-ratios primarily differed significantly between groups on the last day of euthanasia, with ratios being significantly higher in the *E. coli* inoculated groups (Fig. 4C and D). The spleen/BW-ratios were significantly different at one time point between the IT and the Control_1 group, and at 2 dpi and 7 dpi between the Aerosol_2 and Control_2 group. All differences were due to higher ratios in the infected groups.

**Figure 5:**
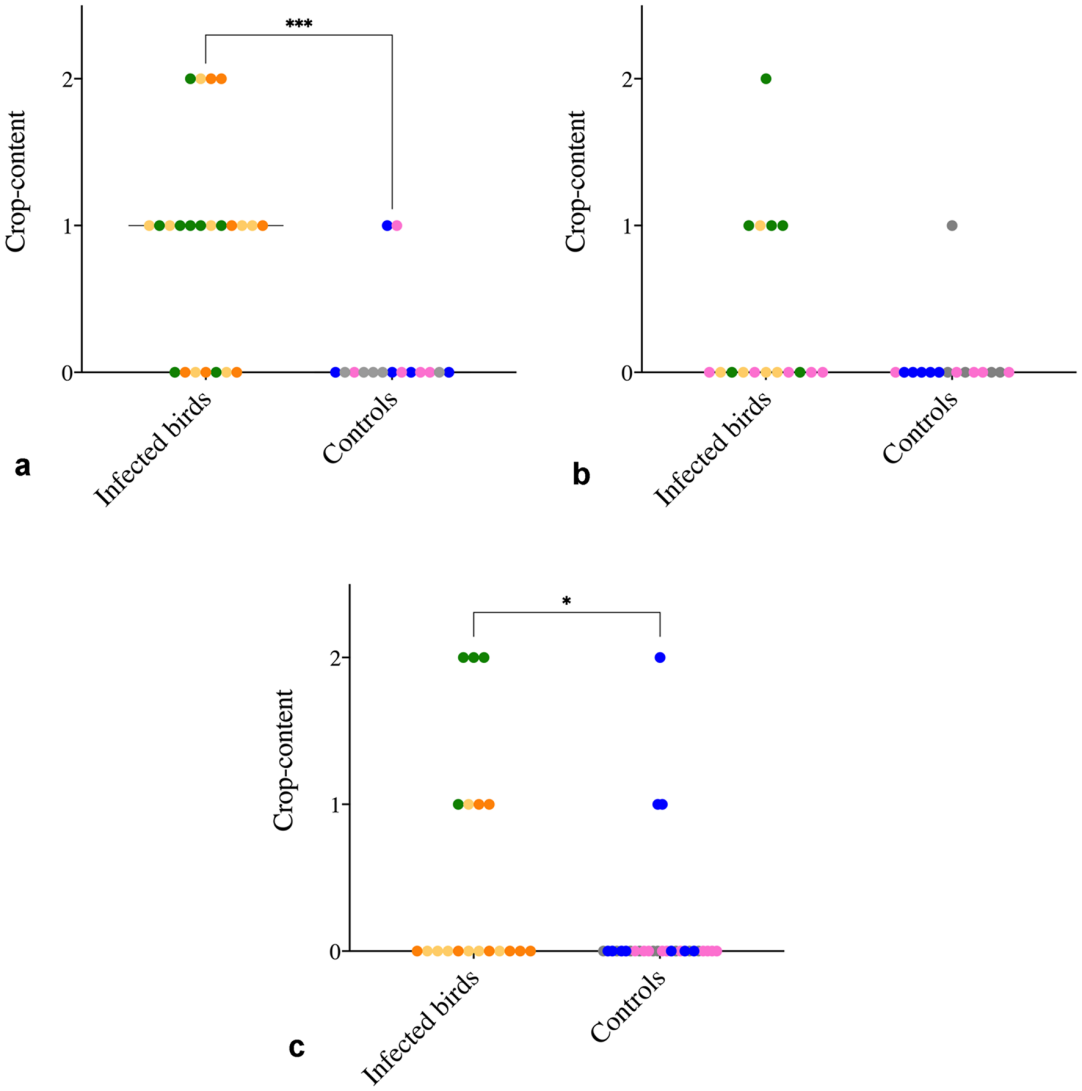
Post-mortem crop-content. Crop-content score in *E. coli*-infected birds vs vehicle-receiving controls at (**a**) 2 dpi, (**b**) 4 and 5 dpi, and (**c**) 6 and 7 dpi. Green, yellow and orange dots represent IT, Aerosol_1 and Aerosol_2, respectively, whilst IT_control is presented with grey dots, Control_1 by pink and Control_2 by blue dots. The absence of palpable content within the crop was graded as 0, presence of noticeable content as 1, and a filled crop as 2. Data is presented with the median and was analysed using a Mann–Whitney test. *p = 0.0269, ***p = 0.0008. *dpi* days post-infection; *IT* intratracheal.

Pooling of the semi-quantitative grading of crop-content revealed a significant difference between infected and control birds at 2 dpi, as the presence of content within the crop was highly linked to being infected with *E. coli* (*p* < 0.001) (Fig. 5). At midpoint euthanasia, a difference could not be established, whereas euthanasia at 6–7 dpi yielded a significant difference between infected and uninfected birds (*p* < 0.05) with none of the remaining IT animals (n = 4) presenting with an empty crop. At necropsy, 13 animals within the IT group (n = 20) were in active lay, defined as having either a developing- or a fully develop egg present within the salpinx, whereas this was the case in all the IT_control animals (n = 19). In both the Aerosol_1 and Control_1 group, 19 animals were in active lay (n = 20 each), whilst 15 met this definition in the Aerosol_2 group (n = 20) and 19 in the Control_2 group (n = 20).

### Microbiology

Bacteriology results from the respiratory organs are presented in Table 6. Sampled sites unrelated to the respiratory tracts (peritoneum, liver, spleen, infundibulum, magnum and the bone marrow), were predominantly negative in all control groups throughout the study, except on few occasions, i.e., at 2 dpi, one Control_2 animal yielded a positive sample from the spleen and another Control_2 bird from the infundibulum part of salpinx, an IT_control bird had a positive peritoneum-sample at 4 dpi, and one Control_2 bird in peritoneum and the spleen at 5 dpi, and, lastly, at 7 dpi, a single bird in the Control_2 group was sampled positive in the peritoneum with a mixed culture dominated by *E. coli*. Amongst the IT inoculated animals, three birds were positive for *E. coli* in all sampled sites (except the bone marrow of one of the birds) at 2 dpi. At 4 dpi, one IT bird was positive in peritoneum, spleen, infundibulum and magnum, whilst another bird was positive in the infundibulum. The surviving IT birds were negative for bacterial growth in all non-respiratory organs at 7 dpi. The IT birds euthanised preterm at 3 dpi were both positive in tracheal swabs, pulmonary, air sac, liver, infundibulum and spleen, while only one of these birds were sampled positive in peritoneum, magnum and the bone marrow also. Amongst Aerosol_1, four birds were positive in all organs at 2 dpi, whereas this was the case for two animals in Aerosol_2. At 4 and 5 dpi, one animal in Aerosol_1 was samples positive in infundibulum and one animal in Aerosol_2 in the spleen, respectively. At 7 dpi, the aerosol inoculated animals were all sterile in all the non-respiratory organs.

**Table 6:** Recovery of *Escherichia coli* from the respiratory organs.

	IT (n = 20)	IT_control (n = 19)^a^	Aerosol_1 (n = 20)	Control_1 (n = 20)	Aerosol_2 (n = 20)	Control_2 (n = 20)
**Trachea**						
2 dpi	5/8	2/5	5/8	1/5	5/7	0/5
3, 4 and 5 dpi	4/7	1/5	1/5	0/5	1/5	1/5
6 and 7 dpi	2/4	0/9	3/7	0/10	0/8	0/10
**Lung**						
2 dpi	7/8	0/5	7/8	0/5	7/7	1/5
3, 4 and 5 dpi	7/7	3/5	4/5	0/5	3/5	1/5
6 and 7 dpi	2/4	0/9	2/7	0/10	0/8	1/10
**Air sacs**						
2 dpi	3/8	0/5	4/8	0/5	2/7	0/5
3, 4 and 5 dpi	3/8	0/5	0/5	0/5	0/5	0/5
6 and 7 dpi	1/4	0/9	0/7	0/10	0/8	0/10

Number of birds positive for *E. coli* in pure culture or with *E. coli* domination at various dpi.

^a^One animal in IT_control was euthanised due to chronic pododermatitis.

*dpi* days post-infection, *IT* intratracheal, *n* number.

A total of 58 isolates re-isolated from chickens in the infection groups (aerosol and IT) were confirmed as *E. coli*. Of these, 40 isolates had a MLVA profile identical to *E. coli* ST117. In the control group, 23 of the re-isolated strains were confirmed as *E. coli* and all, except a single isolate, had MLVA profiles that differed in band patterns from that of *E. coli* ST117.

### Histopathology

**Figure 6:**
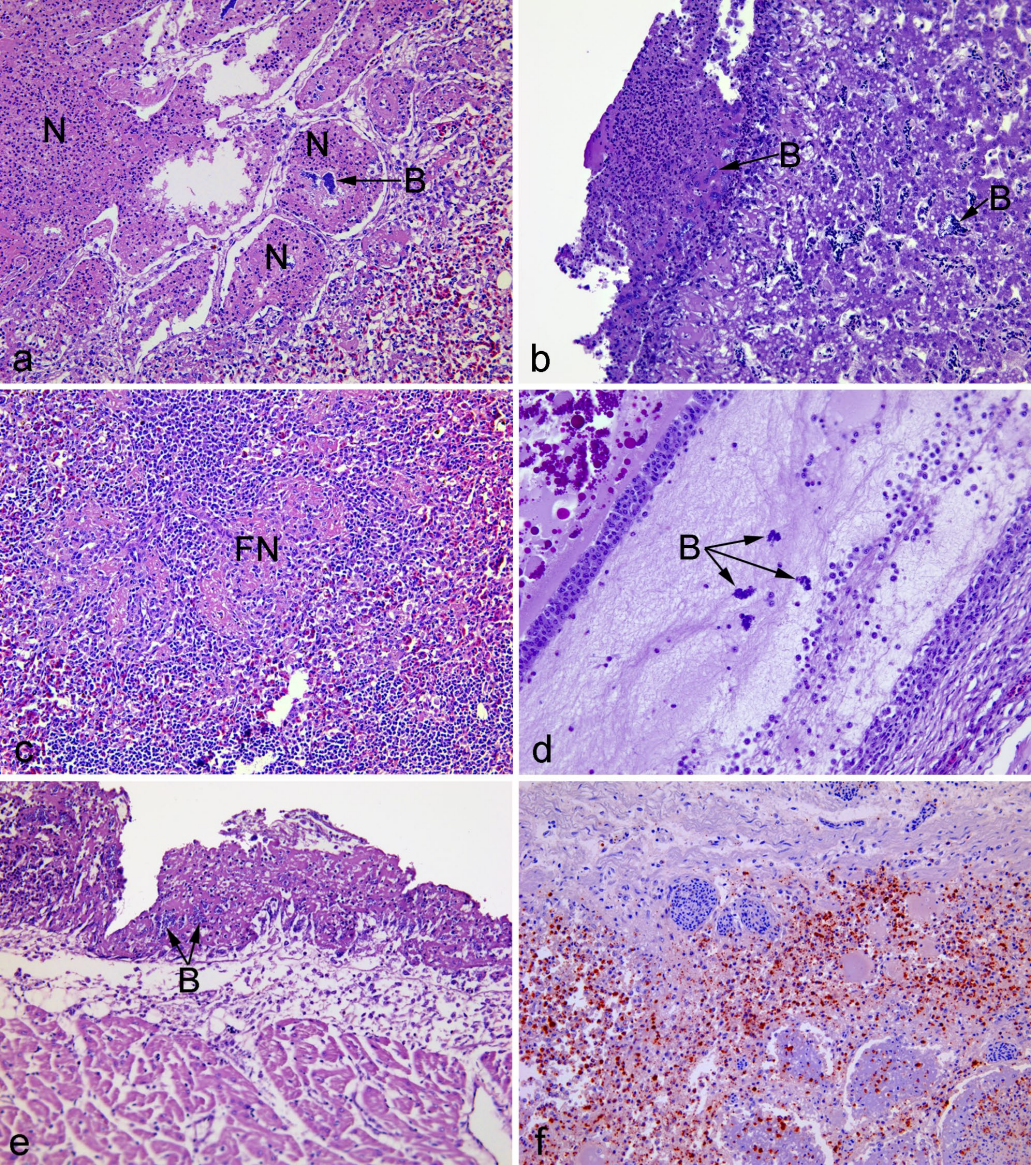
Histopathology of lesions. (**a**) Pulmonary section from an aerosol inoculated animal at 2 dpi displaying prominent masses of fibrinoheterophilic material and necrotic debris within the parabronchial lumen, atriae and infundibulae (N). Intralesional dense-staining bacteria are present (B). The capillary bed appears congested. (**b**) Perihepatitis and subcapsular necrosis in an aerosol inoculated bird. The surface of the liver is covered by a layer of fibrin intermixed with cellular debris and intralesional bacteria (B). A prominent capsular reaction is present with close gathering of histiocytes and lymphocytes. Within sinusoids of the liver, bacteria were easily identified (B). (**c**) Fibrinoid necrosis (FN) in the ellipsoids and periellipsoidal lymphocyte sheath of an aerosol inoculated bird, disturbing the appearance of these structures. (**d**) Folliculitis of an intermediate follicle from an aerosol inoculated bird. The granulosa cell layer is disintegrated and completely separated from the theca interna due to accumulation of proteinaceous fluid and fibrin containing bacterial colonies (B) and sparse amounts of heterophils. (**e**) Pericarditis in an aerosol inoculated bird characterised by a fibrinous mass, necrotic debris and intralesional bacteria (B). The epicardium is expanded by oedema and infiltration by mainly histiocytes. (**f**) Strong immunohistochemical staining of *E. coli* (red-brown) in the lung from an aerosol inoculated bird with necrotic areas in the lung primarily involving the parabronchi and adjacent structures.

In almost all animals, regardless of group affiliation, pulmonary oedema was present to some extent and could be found located both within the lumen of the parabronchi, infundibulae and atriae, as well as in the interstitium and the capillary beds. Areas with necrosis and debris, especially within parabronchi and related structures, were identified in infected animals (Fig. 6A) and, in addition, in some birds belonging to the IT_control group. In the latter, the areas of necrosis were of a modest size. Inflammation was a feature typically demonstrated in infected animals, as was the presence of congested vessels. The latter could also be identified in control groups but to a lesser extent. Blood within airways, as well as plant material, was also noted occasionally in larger airways. Among hepatic sections belonging to the *E. coli* inoculated groups, areas of necrosis, with or without inflammation, were identified in varying degree and both as focal lesions and as more confluent areas. In a subset of infected animals, perihepatitis, as well as occasional subcapsular necrosis, was identified (Fig. 6B). Urate tophi were demonstrated in the hepatic sections of almost half the animals without affiliation to any specific group. Likewise, different degrees of hepatic steatosis were a common finding independent of the group. In splenic sections belonging to the infected groups, fibrinoid necrosis, typically located in the ellipsoids (Fig. 6C), were evident, and in some, distortion of the tissual architecture was also noted. The number of germinal centres in each splenic section varied highly within all groups. A subsection of the infected birds revealed ovarian folliculitis (Fig. 6D) and pericarditis was identified as well (Fig. 6E). Rod-shaped organisms could be identified in some of the H&E-stained sections of all the above tissue types from bacteria-inoculated groups with positive confirmation as *E. coli* by immunohistochemistry (Fig. 6F).

## Discussion

In the present study, a discriminative model of colibacillosis was successfully established by aerosol inoculation of adult broiler breeders. Gross and histopathological lesions characteristic of colibacillosis were produced and were shown to be reproducible at a second time point.

Following a single exposure, aerosol inoculated animals seemed to gradually recover and clear themselves of the challenge strain during the study period. This recovery was supported by the decreasing number of lesions recorded, improvement of feed-digestion and lung/BW-ratio. In contrast, IT inoculated animals did not recover and eventually, all had to be euthanised preterm due to continuing clinical signs and, thereby, animal welfare concerns. Despite continuous respiratory symptoms, half the IT birds did not yield positive bacterial growth at the last euthanasia point, and the remaining were positive only in the respiratory system but not in any other organs. This could indicate permanent damage to the pulmonary tissue as the main cause of continuous clinical signs and not necessarily persistent or progressive *E. coli* infection. The IT_control animals developed minor lung lesions, and at the second point of euthanasia, more than half yielded positive cultures from pulmonary samples, which contained *E. coli,* among other bacteria. This is most likely due to a localised aspiration pneumonia somewhat foreseeable in a model depositing foreign material, even in the case of a sterile vehicle, as a bolus directly into the lungs as this will undoubtedly constitute an irritant. Histopathology supported this view by the presence of relatively restricted areas of necrosis in the pulmonary sections from some of the IT_control birds. A localised, necrotic pneumonia is a well-known consequence of the aspiration of fluids or solid material, likewise is the presence of a mixed population of bacteria^31,32^. Macroscopical changes could not be identified in these birds.

Following *E. coli* exposure, some animals within all the infected groups developed a grossly identifiable purulent and/or fibrinous oophoritis. Likewise, gross changes to the salpinx were recorded in part of the animals. Additionally, both infundibulum and magnum yielded positive bacteriological samples in some of the animals, thereby rendering the risk of vertical transmission highly likely through an aerogenous pathogenesis. Intriguingly, despite clear clinical signs and explicit pathology, many of the birds in the current study were in active lay, defined as having a developing egg present in the oviduct. This, again, underlines the high risk of vertical transmission during such an infectious course.

A higher concentration of *E. coli* in the aerosol chamber was noted at the time of inoculation compared to test runs. This could be due to either the birds contributing to the level of *E. coli* within the chamber through the carriage of dust and faecal material or as a consequence of the movement of the animals within the chamber “stirring” up more *E. coli*. Finally, temperature and/or humidity factors might also have contributed to this difference.

Lung/BW-ratios revealed a significant difference between the infected and the control groups, which was not necessarily detectable upon evaluation of gross pathology, hereby adding a seemingly sensitive and very objective—as well as simple—method to detect differences between the groups in an aerogenous model.

At the last point of euthanasia, hepatic weights were significantly higher in the *E. coli* inoculated birds compared to that of the controls. A feature previously reported in poultry exposed to *E. coli* or LPS derived from *E. coli* potentially attributed to the production of acute-phase reactants^33,34^. Though an increased relative weight of the spleen has formerly been described in colibacillosis^34^ this was only evident in a single aerosol group in the present study, with the relative weight of the spleen generally varying substantially within the groups. Supporting of a lack of difference in relative splenic weight was the absence of follicular hyperplasia in relation to specific groups determined by histopathology.

To improve the model’s construct validity, a lower dose could be provided through a more extended period, eventually resulting in an equal exposure. Alternatively, multiple exposure points could be added. This would better mimic farm conditions with *E. coli* levels being lower than the dose applied in the present study^35^, which were only adhered to due to limitations to the study approval, excluding the opportunity to increase exposure time or apply multiple points of exposure. Still, one should consider the improved air quality in the research facilities, compared to field housing, most likely resulting in the animals being considerably more resistant to respiratory infections as, e.g., ammonia levels represent a known risk factor^10,36,37^. Moreover, the natural exposure to *E. coli* through the air is continuous in the field with dust containing up to approximately 10^5^–10^6^ CFU per gram^37,38^.

Only rather old data from layers on lung volume and respiratory rate were available for calculating the inhaled aerosol dose used in an attempt to dose-match the IT (positive control) to the aerosol groups^39^. Yet, the groups were strikingly similar in respect to gross pathology at the initial day of euthanasia with, e.g., three IT birds presenting with colisepsis, defined as positive growth of *E. coli* in bone marrow samples, whereas four birds in Aerosol_1 and two in Aerosol_2, respectively, had colisepsis at the same time point.

In the present study, the severity of disease was somewhat unexpected, and it can be speculated that heat stress—known to have detrimental effects on poultry^40^—might have contributed to the gravity of disease affecting the animals not only as a stressor but also by altering their respiration towards a heavier and more open-mouth breathing pattern. An improvement to the model, therefore, could be facilitated with appropriate climate control, and replication of the current study must be done with consideration of the heat as a contributing factor to disease severity when deciding on an exposure level.

In the study, a rather forceful method of euthanasia resulted in some damage to tissues in the head area and aspiration of blood, feed, and plant material, which could be disturbing the histopathological evaluation. Refinement of the method of euthanasia could improve animal welfare and assessment of infection outcomes.

In conclusion, a reproducible model of aerogenous *E. coli* infection was established in broiler breeders with close resemblance to spontaneous avian colibacillosis in multiple organs both histologically and grossly. The model holds great potential as a valuable tool for future investigations, especially on prophylactic interventions and disease mechanisms.

## Supplementary Information

Supplementary Information.

## Data Availability

Data, upon which the conclusions in this manuscript relies, are presented within the paper and the supplementary material.

## Abbreviations

AE: Avian encephalomyelitis
BA: Blood agar
BW: Bodyweight
CAV: Chicken anaemia virus
CFU: Colony-forming unit
d: Day
dpi: Days post-infection
*E. coli*: *Escherichia coli*
IB: Infectious bronchitis virus
IBD: Infectious bursal disease
LB: Lysogeny broth
IT: Intratracheal
n: Number
OD: Optical density
W: Week of life
