# Supplementary material for: Development of an aerogenous Escherichia coli infection model in adult broiler breeders
Source: Sci Rep. 2021 Oct 1;11:19556. doi: 10.1038/s41598-021-98270-8 (PMC8486767; doi:10.1038/s41598-021-98270-8)
Supplement: Supplementary file 1 — Supplementary Information. [file 41598_2021_98270_MOESM1_ESM.docx]

| Supplementary table S1: Organ/bodyweight-ratios   \|  \| IT  (n=20) \| IT_control  (n=19)^†^ \| Aerosol_1  (n=20) \| Control_1  (n=20) \| Aerosol_2  (n=20) \| Control_2  (n=20) \| \| --- \| --- \| --- \| --- \| --- \| --- \| --- \| \| BW (g), pre-inoculation \| 3399 ± 204.8 \| 3366 ± 324.5 \| 3450 ± 208.7 \| 3464 ± 168.2 \| 3450 ± 208.7 \| 3511 ± 303 \| \| BW (g), euthanasia \| 3513 ± 320 \| 3520 ± 342.8 \| 3456 ± 259.3 \| 3528 ± 186.9 \| 3483 ± 259.3 \| 3562 ± 334.4 \| \| Lung/BW-ratio  2 dpi \| 0.00358 ± 6.7 × 10^-4*^ \| 0.00241 ± 4.45 × 10^-4^ \| 0.00361 ± 7.45 × 10^-4*^ \| 0.00242 ± 4.34 × 10^-4^ \| 0.00281 ± 3.21 × 10^-4**^ \| 0.002276 ± 1.25 × 10^-4^ \| \| 4 and 5 dpi \| 0.00329 ± 4.84× 10^-4#^ \| 0.00262 ± 3.42 × 10^-4^ \| 0.00276 ± 4.73 × 10^-4^ \| 0.00227 ± 2.75 × 10^-4^ \| 0.00284 ± 3.51 × 10^-4^ \| 0.00275 ± 3.86× 10^-4^ \| \| 6 and 7 dpi \| 0.00337 ± 10.7 × 10^-4^ \| 0.00255 ± 6.58 × 10^-4^ \| 0.0027 ± 6.07 × 10^-4^ \| 0.0026 ± 5.42 × 10^-4^ \| 0.00292 ± 6.41 × 10^-4^ \| 0.00257 ± 6.43 × 10^-4^ \| \| Liver/BW-ratio  2 dpi \| 0.0235 ± 0.00413 \| 0.021 ± 0.00396 \| 0.0274 ± 0.00531^¥^ \| 0.0192 ± 0.003 \| 0.022 ± 0.0046 \| 0.0194 ± 0.0021 \| \| 4 and 5 dpi \| 0.0245 ± 0.003 \| 0.023 ± 0.0068 \| 0.0215 ± 0.0029 \| 0.022 ± 0.00221 \| 0.0268 ± 0.0033 \| 0.0231 ± 0.00343 \| \| 6 and 7 dpi \| 0.0349 ± 0.00332^a, b^ \| 0.0247 ± 0.00203 \| 0.0271 ± 0.0042^¥^ \| 0.22 ± 0.00338 \| 0.03 ± 0.00483^«^ \| 0.0251 ± 0.0026 \| \| Spleen/BW-ratio  2 dpi \| 0.001 ± 2.828 × 10^-4^ \| 0.000745 ± 1.51 × 10^-4^ \| 0.000832 ± 1.44 × 10^-4^ \| 0.000749 ± 1.46 × 10^-4^ \| 0.000856 ± 1.68 × 10^-4«^ \| 0.000638 ± 9.31 × 10^-5^ \| \| 4 and 5 dpi \| 0.000971 ± 1.16 × 10^-4¥^ \| 0.000727 ± 1.37 × 10^-4^ \| 0.000731 ± 1.17 × 10^-4^ \| 0.000698 ± 9.8 × 10^-5^ \| 0.000955 ± 1.66 × 10^-4^ \| 0.000768 ± 1.71 × 10^-4^ \| \| 6 and 7 dpi \| 0.000831 ± 2.04 × 10^-4^ \| 0.000817 ± 1.52 × 10^-4^ \| 0.00077 ± 6.03 × 10^-5^ \| 0.000857 ± 2.12 × 10^-4^ \| 0.00114 ± 0.00666^«^ \| 0.000659 ± 1.07 × 10^-4^ \| |
| --- | --- | --- | --- | --- | --- | --- | --- | --- | --- | --- | --- | --- | --- | --- | --- | --- | --- | --- | --- | --- | --- | --- | --- | --- | --- | --- | --- | --- | --- | --- | --- | --- | --- | --- | --- | --- | --- | --- | --- | --- | --- | --- | --- | --- | --- | --- | --- | --- | --- | --- | --- | --- | --- | --- | --- | --- | --- | --- | --- | --- | --- | --- | --- | --- | --- | --- | --- | --- | --- | --- | --- | --- | --- | --- | --- | --- | --- | --- | --- | --- | --- | --- | --- | --- |
| ^†^ One animal in the IT_control group was euthanised due to a chronic pododermatitis unrelated to the study. Data are presented as mean ± standard deviation. ^*^ *p < 0.01* compared to Control_1 and IT_control. ^**^ *p < 0.01* compared to Control_2. ^#^ *p < 0.01* compared to Control_1. ^¥^ *p < 0.05* compared to Control_1.  ^a^ *p < 0.0001* compared to IT_control and Control_1.  ^b^ *P < 0.01* compared to Aerosol_1. ^«^ *p < 0.05* compared to Control_2. The lung/BW-ratio and liver/BW-ratio from IT, IT_control, Aerosol_1 and Control_1, and the bodyweight comparisons, were analysed using ANOVA and Tukey’s multiple comparison test. Likewise, these tests were used to analyse the spleen/BW-ratio of these groups, except for dpi 4, as the data did not follow a normal distribution at this timepoint. Therefore, a Kruskal-Wallis test and Dunn’s multiple comparisons test were applied instead. Aerosol_2 and Control_2 were compared using a T-test except when comparing the spleen/BW-ratio at dpi 7 due to deviation from a normal distribution. Instead, a Mann-Whitney test was used. Abbreviations: BW, bodyweight; IT, intratracheal; n, number. |
